# How researchers calculate students’ grade point average in other courses has minimal impact

**DOI:** 10.1371/journal.pone.0290109

**Published:** 2023-08-18

**Authors:** Nicholas T. Young, Rebecca L. Matz, Eric F. Bell, Caitlin Hayward

**Affiliations:** 1 Center for Academic Innovation, University of Michigan, Ann Arbor, Michigan, United States of America; 2 Department of Astronomy, University of Michigan, Ann Arbor, Michigan, United States of America; Central Queensland University, AUSTRALIA

## Abstract

Grade point average in “other” courses (GPAO) is an increasingly common measure used to control for prior academic performance and to predict future academic performance. In previous work, there are two distinct approaches to calculating GPAO, one based on only courses taken concurrently (term GPAO) and one based on all previous courses taken (cumulative GPAO). To our knowledge, no one has studied whether these methods for calculating the GPAO result in equivalent analyses and conclusions. As researchers often use one definition or the other without comment on why that choice was made, if the two calculations of GPAO are different, researchers might be inducing systematic error into their results and publishing potentially inaccurate conclusions. We looked at more than 3,700 courses at a public, research-intensive university over a decade and found limited evidence that the choice of GPAO calculation affects the conclusions. At most, one in seven courses could be affected. Further analysis suggests that there may be situations where one form of GPAO may be preferred over the other when it comes to examining inequity in courses or predicting student grades. However, we did not find sufficient evidence to universally recommend one form of GPAO over the other.

## Introduction

Colleges and universities face pressure from students, parents, politicians, and the public to demonstrate positive educational outcomes such as high retention and graduation rates. It is then unsurprising that prediction of student performance and likelihood of dropping out are two of the most common research areas in the learning analytics and educational data mining communities [1, 2], often with the goal of implementing supportive interventions. As grades have a central role in determining whether a student will stay in their major or even at the university [3–9], many of these efforts focus on predicting how a student will perform in a course or on an exam in the course. Typically, these studies include some measure of students’ prior academic performance such as high school grade point average (GPA) or standardized test scores to control for preparation or as a predictor in the model [10–15].

In recent years, researchers have started to use a metric called grade point average in “other” courses (GPAO), meaning the average grade in other courses taken at the institution with the exception of the course of interest, to account for academic performance in a specific course in retrospective analyses [16–18] as it has been found to be more predictive of course grades and final exam grades than high school GPA and SAT/ACT scores [19, 20]. As Huberth et al. note, this finding is unsurprising because unless one course is “utterly unlike” other courses at an institution, we would expect that performance in one course should inform how the student will do in other courses at the institution [16].

Yet, there have been alternative interpretations in the literature as to what “other” courses means. One approach to GPAO, which we call *cumulative GPAO*, uses grades from all other courses taken up to and including the term of interest at the university in the calculation [16, 20–28]. Alternatively, other studies have calculated GPAO based only on the grades of the courses the student is enrolled in during the same term as the course of interest [17, 29, 30]. We refer to this approach as *term GPAO* because only courses from the same term as the course of interest are included.

Although the difference may seem pedantic, the methods we as researchers use can influence our findings, and therefore we should reflect on the detailed elements of the methods we choose [31]. As GPAO can be used for educational equity analyzes, such as to examine whether courses, programs, and environments are equitable for all students, such as Matz et al. [20], knowing whether the definition of GPAO influences the findings researchers and administrators use to allocate limited institutional resources for interventions is important and can help prevent resources from being misallocated. As recent work has called for researchers to account for limitations that exist in assessing students’ prior performance [32], research should be done into where they may lie with GPAO. This study attempts to fill this gap and provide an evidence-based understanding of GPAO measures.

Based on the definitions of the two GPAO measures, there are reasons why one might be preferred over the other, and the differences (and outcomes) should be examined. For example, as a result of using all courses past and concurrent, the cumulative GPAO method is less sensitive to the grade in a single course and thus, will be less variable than the term GPAO method. However, term GPAO may be better able to account for non-academic events such as physical and mental illness, care-taking responsibilities, relationship or personal issues, or world events that could have affected a student’s performance in a temporally-bound way that might be obscured by the cumulative GPAO, as well as academic factors such as grade inflation [33–35], or navigating a particularly challenging courseload. Term GPAO may also be more useful for analyses conducted in advanced courses or with advanced students because prior performance in large-enrollment, “weed-out” introductory courses may not be representative of performance in advanced courses, and students’ GPAs often increase after their first term [36, 37]. In either case, however, the GPAO can only be calculated after the term of interest is complete.

For this study, we focus on the two definitions of GPAO, the difference between a student’s GPAO and their final grade in the class of interest, the grade anomaly [20], the type of course, and the identity of the student based on selected demographic characteristics. Specifically, we ask four research questions:

- RQ1: How does the term GPAO compare to the cumulative GPAO and, by extension, term grade anomaly to cumulative grade anomaly?
- RQ2: How does the answer to the above question change based on whether the course is an introductory, intermediate, or advanced level course? How does the answer change based on whether the course is a lab or lecture course?
- RQ3: How do the answers to the above questions change when breaking results down by demographics?
- RQ4: How does a researcher’s choice of using the term GPAO or the cumulative GPAO in the grade anomaly calculation affect conclusions about the course?

This is a comparison study; as such, we follow the criteria laid out by Boulesteix et al. [38], which are discussed in S1 File. These criteria provide a productive frame given that comparison studies “may be necessary to ensure that previously proposed methods work as expected in various situations and that emerging standard practice rules adopted by substantive researchers or statistical consultants are the result of well-designed studies” [38]. Our goals are then to document the affordances and constraints of two different methods for calculating GPAO, whether one definition should be preferred over the other, and best practices for using GPAO.

## Materials and methods

### Mathematical definitions of GPAO and grade anomaly

First, we briefly provide formal definitions of cumulative GPAO, term GPAO, cumulative grade anomaly, and term grade anomaly.

For a student enrolled at a university, let *x*_*ij*_ be the numeric grade earned by them in their *j*th course taken during their *i*th term enrolled at the university and *c*_*ij*_ be the number of credit hours of the *j*th course taken during the students’ *i*th term. The cumulative GPAO for this student for a specific course (denoted j*) taken during their i*th term is then given by

$$\begin{array}{c} \text{cumulative}\;{\text{GPAO}}_{i^{*},j^{*}}=\frac{\sum_{i\leq i^{*},j\neq j^{*}}c_{ij}x_{ij}}{\sum_{i\leq i^{*},j\neq j^{*}}c_{ij}} \end{array}$$

and their term GPAO is given by

$$\begin{array}{c} \text{term}\;{\text{GPAO}}_{i^{*},j^{*}}=\frac{\sum_{i=i^{*},j\neq j^{*}}c_{ij}x_{ij}}{\sum_{i=i^{*},j\neq j^{*}}c_{ij}} \end{array}$$

As the grade anomaly is the difference between the grade earned in the course and the student’s GPAO, the cumulative and term grade anomalies for the specific course (again denoted j*) taken during the student’s i* term are

$$\begin{array}{c} \text{cumulative}\;{\text{anomaly}}_{i^{*},j^{*}}=x_{i^{*},j^{*}}-\frac{\sum_{i\leq i^{*},j\neq j^{*}}c_{ij}x_{ij}}{\sum_{i\leq i^{*},j\neq j^{*}}c_{ij}} \end{array}$$

$$\begin{array}{c} \text{term}\;{\text{anomaly}}_{i^{*},j^{*}}=x_{i^{*},j^{*}}-\frac{\sum_{i=i^{*},j\neq j^{*}}c_{ij}x_{ij}}{\sum_{i=i^{*},j\neq j^{*}}c_{ij}} \end{array}$$

respectively.

### Data collection

Our data comes from the student data warehouse at a large, public, research-intensive university. This data set provides deidentified information about students, including course enrollments, grades earned, majors and minors, demographics, and prior educational experiences, among other information. Grades were recorded in letter form (e.g. A, B-, etc.) and we converted them to the standard 4.0 scale, with an A being a 4.0, an A- a 3.7, a B+ a 3.3, and so on, for this study. Both term and cumulative GPAO values are provided in the dataset for each grade outcome observed (with some exceptions—detailed below). For this paper, we looked at grades and GPAOs for all undergraduate course enrollments from Fall 2009 through Fall 2019, including summer terms, for students who enrolled in Fall 2009 or later. We removed course records for any students who took the course during their first semester at the university from our analysis because, by definition, the term GPAO and cumulative GPAO are equivalent in this case. This choice removed 16% of the student course records in the original data set. We then removed any course that did not have at least 50 students enrolled during the time period to eliminate any one-off courses or courses with very low enrollment (50 students in the data set correspond to at least 5 students taking the course per calendar year), resulting in nearly 60% of the course offerings being dropped but only 4% of the remaining student records. After doing so, we were left with 3737 usable course offerings, corresponding to about 1.85 million student records. Student records without GPAOs were only dropped for specific analyses using each GPAO. For example, if a student was missing term GPAO but had a cumulative GPAO, their record would be dropped from the median term GPAO calculation but not the median cumulative GPAO calculation. This case is possible if a student took only one course in a given term or withdrew from all of their other courses during a term and occurred for at most 1.6% of student records.

### Data analysis

For each course, we calculated the median term and cumulative GPAOs. Because we had access to students’ final course grades, we also calculated the median term and cumulative grade anomalies, which are the median differences between each student’s final grade and GPAO (because the median is not additive, it is important to note that this calculation is not equivalent to the difference between the median final grade and the median GPAO). A positive grade anomaly (“grade boost”) means that the student earned a higher grade in the course of interest relative to their performance in “other” courses while a negative grade anomaly (“grade penalty”) means that the student earned a lower grade in the course of interest relative to their performance in “other” courses. The median anomalies then determine whether a “typical” student (i.e., a student with a grade anomaly in the 50th percentile) earned a grade boost or a grade penalty in the course. Analyses using the means instead of the medians are included in S2 File.

Because the central tendency of the grade anomaly distributions could hide differences in the tails of the distributions, we also looked at how many grade anomalies exceeded a certain threshold, *θ*, in magnitude, which we picked to be either 0.7 or 1.0 grade points. We chose this range because for a grade anomaly to be this large or greater (*anomaly* ≥ *θ*), the grade earned in a course would have to be at least one letter or two signs different from the GPAO, indicating a relatively large error from a prediction standpoint (e.g., the student had a GPAO of 3.7, A-, but earned a grade of 3.0, B). We calculated the fraction of students with a final grade more than *θ* = 0.7 and *θ* = 1.0 away from their term GPAO and cumulative GPAO, respectively, in each course. We then counted the number of courses where using the term GPAO resulted in more students beyond the threshold than using the cumulative GPAO and vice versa for each threshold. Such a result would be useful to know in the case that the GPAO is used in a model to predict a student’s final grade in a course.

### Demographic analysis

Due to the increased attention to equity and demographic gaps in education research [39, 40], we also examined how the GPAOs compare across groups of students with different selected demographic identities. If GPAO is to be used as a measure of prior performance or to control for prior performance in regression analyses, it is important to know whether the measures are equivalent for all students or if there are situations where using one GPAO instead of the other could systematically affect a result. For example, if a researcher is using grade anomalies to determine whether a course is equitable for students of different demographic groups, it would be problematic if using the term GPAO resulted in the conclusion that the course offered a grade boost to students while using the cumulative GPAO resulted in the conclusion that the course offered a grade penalty.

For this analysis, we focused on five demographic categories that are often associated with grade differences and are included in our student data warehouse. As the data come from the data warehouse and are not collected by us, we had no control over what options students could select to describe their identity. The five categories were sex, race, socioeconomic status, first-generation status, and transfer status. Sex was treated as a binary variable (male or female) even though sex is not binary [41] and gender is the construct of greater interest. Race consisted of five options, Asian, Black, Hispanic, Native American, and White, and students who selected more than one of the five options were marked as multi-racial. We then collapsed these data into an underrepresented minority category (Black, Hispanic, multi-racial, and Native American) and a non-underrepresented category (Asian and White) to ensure we had sufficient students in each group to detect any trends. Given that the underrepresented minority label is considered racist and harmful [42–44], we will instead refer to these groups as “B/H/M/N” and “A/W”, using the first letter of the term that students used to identify themselves. We acknowledge that such groupings obscure the unique situations of each group and mask the struggles of individuals [42, 45, 46]. However, as using a binary grouping of race is relatively common in educational research, we believe that our study should replicate the structure of the data that researchers themselves are using to greatest degree possible. As such, we also did not split the race variable by international status; we acknowledge that there are likely to be differences between domestic and international students identifying as the same race. We defined first-generation status as a binary variable in which we marked any student whose parents did not complete at least a 4-year degree after high school as a first generation student. We considered a student as low income if the median annual household income of the student’s high school’s zip code was less than $60,000. We used this metric rather than the student’s self-reported estimated family income due to a large amount (more than 20%) of missing data. We conservatively coded students without a zip code (such as international students) as not low income. We considered transfer students as those who did not enter the university as a first-time college student. Students with missing data were only excluded from the analysis for the specific demographic variable that they were missing.

To determine whether our choice of GPAO might affect conclusions about equity gaps, we computed the median term anomaly and the median cumulative anomaly in all of the courses split by our ten demographic categories (male students; female students; Asian and White students; Black, Hispanic, multi-racial, and Native American students; low income students; non-low income students; first-generation students; continuing-generation students; transfer students; and non-transfer students). We identified where our choice of GPAO could impact the conclusions as cases when there were at least 50 students in the demographic group in the course over the 10-year period, one of the median grade anomalies was positive and the other was negative, and at least one of the median grade anomalies was at minimum 0.099 grade points to ensure that we were unlikely to be accounting for differences due to intrinsic variability in the data.

To determine the impact of our choice of the minimum grade point difference on the results, we repeated the analysis using 0.049 instead of 0.099 as the minimum difference needed to indicate a conclusion changed.

### Subset of courses

While summary statistics such as the median are useful to understand the general trend, they may mask important information hidden in the distribution of the data. To investigate this, we conducted additional analysis on a subset of 18 STEM (science, technology, engineering, and mathematics) courses informed by our previous work using GPAO and the type of courses studied in previous GPAO papers. Additionally, we focused on STEM courses because they often assign lower grades than non-STEM courses [47, 48] and have documented grade inequities [37, 49]. We varied these courses along three parameters: discipline (biology, chemistry, and physics), level (introductory, intermediate, and advanced), and format (lecture and lab). The lab courses we selected require prior or concurrent enrollment with the lecture course. The courses, along with the level, the number of students enrolled over the period of the study and included in the analysis, and the average grade of those students in the course, appear in Table 1.

**Table 1:** The courses, their level, number of students included in the analysis, and average final grade of those students.

Course name	Level	N	Average Grade
Intro biology	Introductory	8,762	2.922
Intro biology lab	Introductory	12,752	3.385
Human & animal physiology	Intermediate	5,410	3.038
Human & animal physiology lab	Intermediate	4,231	3.680
Genetics	Advanced	6,026	2.799
Genetics lab	Advanced	1,297	3.348
General chemistry	Introductory	7,984	2.784
General chemistry lab	Introductory	7,261	3.256
Organic chemistry	Intermediate	12,405	2.774
Organic chemistry lab	Intermediate	11,489	3.653
Physical chemistry	Advanced	248	3.246
Physical/Computational chemistry lab	Advanced	231	3.773
Physics 1	Introductory	8,753	2.735
Physics 1 lab	Introductory	9,001	3.312
Physics 2	Intermediate	8,502	2.883
Physics 2 lab	Intermediate	9,669	3.505
Intro modern physics	Advanced	974	3.261
Intro modern physics lab	Advanced	512	3.614

First, we repeated the analysis examining differences in the tails of the grade anomaly distributions. This time, we were interested in whether the differences were statistically and practically different. We again looked at how many grade anomalies exceeded a certain threshold, *θ*, in magnitude, this time ranging between 0.7 and 1.0 grade points to examine in greater depth how our choice of *θ* influenced the results. We determined whether the difference mattered statistically by a difference of proportions test. To test for practical significance, we adopt the criteria that for the difference to matter, the difference should be at least 5 percentage points. If there are differences between the two GPAOs at the tails of the distribution, we would expect that using either term or cumulative GPAO would result in more students having grade anomalies above the threshold than the other.

Next, we examined differences between the two GPAOs in the subset of courses. We investigated this question in two ways, covering two methods in which GPAO might be used. First, we used the Wilcoxon signed-rank test to compare the two GPAO measures. We do so because we are interested not only in whether there is a difference between the two GPAO measures but also the direction of the difference. This approach is equivalent to comparing the GPAO methods at the student level, and the results would be applicable if we were interested in predicting a student’s grade from their GPAO. Second, we used the median test to compare the median term GPAO and the median cumulative GPAO in each of the courses. This approach is equivalent to comparing the GPAOs at the course level and would be useful for comparing outcomes across courses. We then repeated the median tests for the term anomaly and the cumulative anomaly in each of the courses. As the difference between the two GPAOs and the difference between the two grade anomalies is equivalent up to a sign at the student level (that is, *anomaly*_*term*_ − *anomaly*_*cum*_ = (*grade* − *GPAO*_*term*_) − (*grade* − *GPAO*_*cum*_) = −(*GPAO*_*term*_ − *GPAO*_*cum*_)), we only perform the Wilcoxon tests on the two GPAO measures and not the two anomaly measures. Due to increasing calls to move away from solely relying on statistical significance and p-values to determine conclusions [50], we also calculated the effect size using the Wilcoxon Effect Size, *r* [51]. We consider *r* ∈ [0.1, 0.3) as a small effect, *r* ∈ [0.3, 0.5) as a medium effect, and *r* ∈ [0.5, ∞) as a large effect [52]. For the three analyses, we performed them both for all students in the course and for only students in each of the ten demographic categories identified in the previous section.

Because we are comparing GPAOs for multiple demographic groups and hence conducting multiple tests of statistical significance, we used the Holm-Bonferroni procedure to control for multiple tests within each individual course. We control within courses rather than across courses for two reasons. First, for our results to be applicable to future studies, we want our study to match as closely as possible to what researchers do. Second, following the criteria of Boulesteix et al. [53], we are discussing and reporting the results of each course regardless of whether we find statistical differences but placing emphasis on statistically significant results within courses. Therefore, we should control within courses but not across courses. More formally, we are considering the results within each course to be a family and therefore, we should control the *p*-values within each course [54].

## Results & discussion

In this section, we begin by zooming in on GPAO and grade anomalies in the subset of 18 courses to first gain an in-depth of understanding of GPAO and grade anomaly. We then zoom out to the full institutional level, leveraging our understanding from the subset of courses.

### Subset of courses

**Fig 1:**
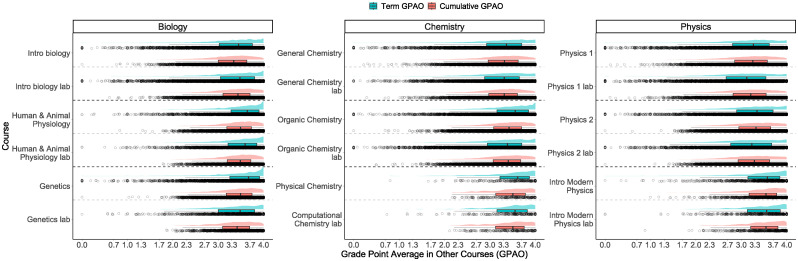
Distribution of the term (blue) and cumulative (red) GPAOs in the selected 18 courses. In general, the median term GPAO tends to be higher than the median cumulative GPAO and the term GPAO distribution tends to have a peak around 4.0 while the cumulative GPAO distribution does not.

From Fig 1, we find that in many of the courses, there is a relatively large number of students with a term GPAO of 4 but very few students with a cumulative GPAO of 4.0 (e.g., Intro biology, Human & animal physiology and Organic chemistry in Fig 1).

Second, the tails of the term GPAO distributions in all courses tend to be longer than those of the cumulative GPAOs. This likely reflects the fact that fewer data points (grades) are considered in the term GPAO calculation than the cumulative GPAO calculation and hence, it is harder for higher grades to balance out the lower grades. Breaking the results down by the type of course, we find that the tails of the distributions in advanced chemistry and physics courses are smaller than the introductory courses. The same is true for the advanced biology courses but only for the cumulative GPAO. The distributions between the lecture and lab course at each level and in each discipline appear to be roughly similar.

Finally, we find that the patterns of which GPAO is larger depend on the course’s format. In all nine of the lecture courses, regardless of the discipline, the term GPAO is larger than the cumulative GPAO. Alternatively, the term GPAO is only larger in five of the nine lab courses.

**Fig 2:**
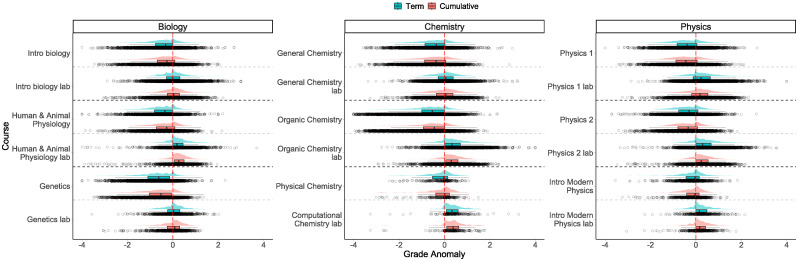
Distribution of the term and cumulative grade anomalies in the selected 18 courses. In general, the median cumulative grade anomaly is closer to zero, meaning that the median difference between the student’s grade and GPAO is smaller when the cumulative GPAO is used.

The grade anomalies show a similar result (Fig 2). First, for all introductory and intermediate lecture courses and the advanced biology course, the grade anomalies are negative, while for laboratory courses, the anomalies are either positive or centered around zero. In contrast, in the advanced physics and chemistry courses, the grade anomalies are centered around zero. We also notice that the distributions are smaller in this case. These negative grade anomalies in lower- and intermediate-division lecture courses mean that students do worse in these courses compared to their “other” courses at the university.

Looking at the values of median grade anomalies, we find that the median term grade anomaly and median cumulative grade anomaly are mostly within 0.1 grade points of each other and in general, agree with the direction of the effect (i.e., a grade boost or grade penalty). We find this to be true regardless of the discipline, level, or format of the course.

Considering the magnitude of the anomalies, we find that the median cumulative anomaly is closer to zero than the median term anomaly in 10 of the 18 courses.

### Outlier analysis

Our results regarding the fraction of students in each course with a grade anomaly outside of some threshold *θ* are shown in Table 2. If the tails of the grade anomaly distributions are different, we would expect to see a difference in the fraction of students beyond each *θ*.

**Table 2:** Percentage of students with a term or cumulative grade anomaly outside of the threshold *θ*. A * means that the difference is statistically significant (*p* < 0.05).

Course	Term *θ* = 0.7	Cum *θ* = 0.7	Δ *θ* = 0.7	Term *θ* = 0.8	Cum *θ* = 0.8	Δ *θ* = 0.8	Term *θ* = 0.9	Cum *θ* = 0.9	Δ *θ* = 0.9	Term *θ* = 1.0	Cum *θ* = 1.0	Δ *θ* = 1.0
Intro biology	31.7	26.9	4.8*	25.5	22.1	3.4*	20.6	17.9	2.7*	17.5	14.9	2.6*
Intro biology lab	16.5	10.3	6.2*	11.4	6.9	4.5*	8.5	4.7	3.8*	6.5	3.2	3.3*
Human & animal physiology	33.7	28.9	4.8*	26.7	23.7	3*	22.6	19.2	3.3*	19.1	16.2	2.9*
Human & animal physiology lab	15.7	12.8	2.8*	11.5	8.5	3*	8.7	5.8	2.8*	6.6	4.2	2.5*
Genetics	47.2	40.9	6.4*	40.0	35.5	4.5*	35.2	30.7	4.5*	30.9	27.0	3.8*
Genetics lab	17.0	12.8	4.2	12.1	9.4	2.7	7.9	7.2	0.7	6.6	5.2	1.4
General chemistry	36.2	34.7	1.5	29.8	29.8	-0.1	25.2	25.2	0	21.6	21.4	0.2
General chemistry lab	19.5	20.4	-0.8	14.2	14.3	-0.1	10.1	10.1	0	7.3	6.9	0.4
Organic chemistry	42.7	37.2	5.5*	36.2	31.7	4.5*	31.3	27.4	4*	27.0	23.7	3.4*
Organic chemistry lab	27.9	19.9	8*	22.2	14.4	7.8*	18.1	10.5	7.7*	14.8	7.6	7.2*
Physical chemistry	21.0	16.1	4.8	15.7	14.5	1.2	12.9	11.7	1.2	10.1	9.7	0.4
Physical & Computational chemistry lab	22.1	20.8	1.3	19.5	17.3	2.2	16.0	13.4	2.6	13.9	12.1	1.7
Physics 1	35.6	37.4	-1.8	29.3	31.1	-1.8	24.1	25.3	-1.2	19.8	20.3	-0.5
Physics 1 lab	27.5	23.9	3.7*	22.1	18.3	3.7*	17.9	13.7	4.2*	14.2	10.0	4.1*
Physics 2	30.8	31.6	-0.8	25.1	25.7	-0.7	20.4	20.1	0.3	16.2	15.4	0.8
Physics 2 lab	26.0	16.5	9.5*	20.3	11.8	8.5*	16.0	8.7	7.3*	13.1	6.2	6.9*
Intro modern physics	18.1	13.4	4.6	13.2	10.5	2.8	10.0	7.6	2.4	7.5	5.5	2
Intro modern physics lab	18.8	13.5	5.3	14.6	9.6	5.1	10.9	7.2	3.7	8.0	5.3	2.7

For all the biology courses except for genetics lab, we do find that to be the case. While these differences are only a small percentage of students, the term grade anomaly does seem to have larger tails for most of the biology courses, regardless of which threshold we use. We find the same to be true for the organic chemistry courses, as well as physics 1 lab and physics 2 lab.

In the other courses, we do not find such differences. In some cases, changing the threshold value *θ* changes which GPAO results in more students beyond the threshold (e.g., general chemistry lab and physics 2). We note that even if the difference in the fraction of students is not different between the two GPAO measures, there can still be a relatively large number of students beyond the threshold. For example, regardless of which GPAO we use, about 15% of the students in physics 2 earned a grade that was more than 1 grade point different from their average grade in their “other courses” (GPAO) while genetics had the highest fraction of students outside of the 1 grade point threshold at more than a quarter of students. Using our practical significance cutoff of at least 5 percentage points different, six courses would be practically significant for *θ* = 0.7 and two courses would be practically significant for *θ* = 1.0.

### Demographics analysis

**Fig 3:**
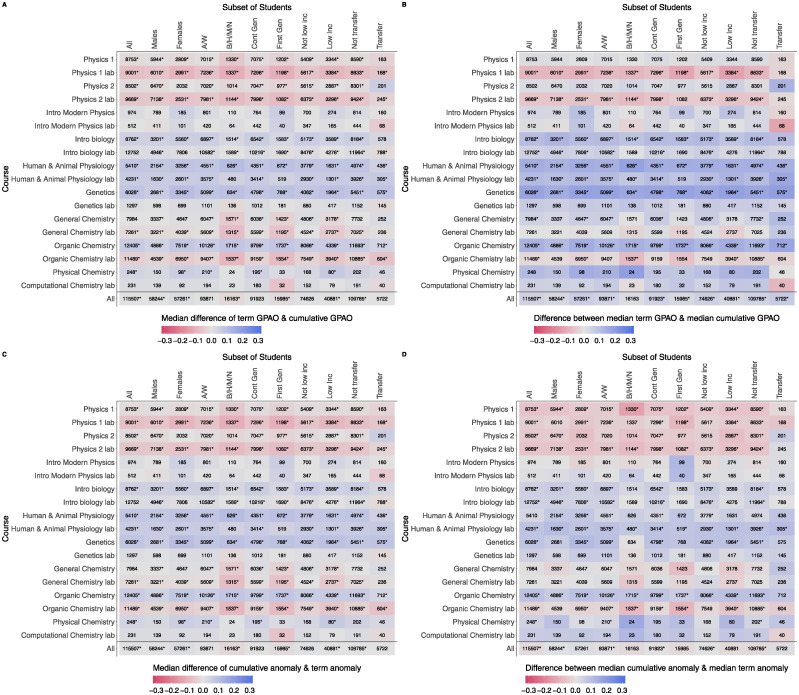
The difference between the term GPAO and cumulative GPAO in each course (3A and 3B) and the difference between the cumulative anomaly and term anomaly in each course (3C and 3D), split by demographic groups. In the top plots, red signifies that the cumulative GPAO is larger and blue signifies the term GPAO is larger. In the bottom plots, blue indicates the cumulative anomaly is larger while red indicates the term anomaly is larger. The sample size is represented by the number in each cell, with “*” signifying that a Wilcoxon test (left plots) or a median test (right plots) found the difference to be statistically significant. The left plots show the median differences computed at the student level while the right plots show the differences of medians computed at the course level. While we find consistent patterns within courses, we do not find a consistent pattern across courses.

While the previous results considered GPAOs and grade anomalies at the course level, it is also important to consider possible differences at the student level. The results of doing so with the Wilcoxon tests overall and by demographic variables at the student level are shown in Fig 3A and the results of the median tests overall and by demographic at the course level are shown in Fig 3B. We do not find a consistent pattern across all the courses studied here, but we do find that the patterns tend to be consistent within each course. For example, we notice that students in physics 1 and 2 labs have a larger cumulative GPAO than term GPAO while students in the intermediate and advanced biology courses tend to have a larger term GPAO. The results also show that the trends are consistent regardless of whether the analysis looks at the differences at the student level or the course level. However, the differences tend to be larger at the course level than the student level. In addition, when aggregating across all students at the course level, the median term GPAO is larger than the median cumulative GPAO for all demographic groups.

Looking at the specific demographics, we find there are cases where there is a statistically significant difference between the two GPAO measures. For example, in nearly all of the introductory and intermediate level courses, there is a statistical difference between the two GPAOs for male students at the student level. However, the direction of the effect is not consistent across courses. Yet, there was no demographic group where a statistical difference was always found at either the student or course level. We also do not find a consistent result in terms of which GPAO is larger across courses in a discipline with the exception of the biology courses in which students tend to have larger term GPAOs, both at the student and course level of analysis. Nevertheless, even though the size of the difference is not statistically significant, it is important to note that the two GPAOs measures do produce different impacts on the demographic groups.

Fig 3C and 3D show the results of the analysis for the grade anomalies. As noted with the GPAOs, there is not a consistent pattern for each demographic group across the courses. In some cases, like the biology courses, the direction of the effect is consistent but the difference is not always statistically significant. Likewise, the direction of the effect is mostly consistent within each course as was the same with the GPAOs. However, when aggregating across students, we do not see a consistent trend. Unlike for the GPAOs, only the largest student groups show a statistically significant difference.

**Fig 4:**
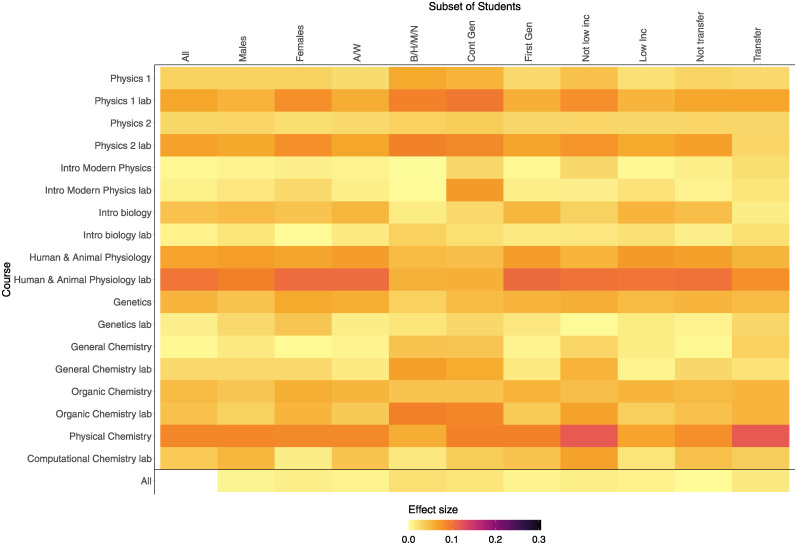
The effect sizes of the differences between the term GPAO and cumulative GPAO in each course, split by demographic groups. A darker color signals a larger effect size. For most demographic groups in most courses, the effect size is considered trivial (< 0.1) while in a few cases, it is considered small.

Finally, the effect sizes show similar results (Fig 4). There are a few groups in a few courses that have a small effect size such as female students, Asian and White students, first-generation students, and non-transfer students in human and animal physiology lab, but most demographic and course combinations have a trivial effect size (*r* < 0.1). The same is true for student groups when aggregating across all courses.

### All courses

Building on our results from the subset of courses, we now change our focus to all courses with at least 50 enrolled during the time period studied at the institution.

**Fig 5:**
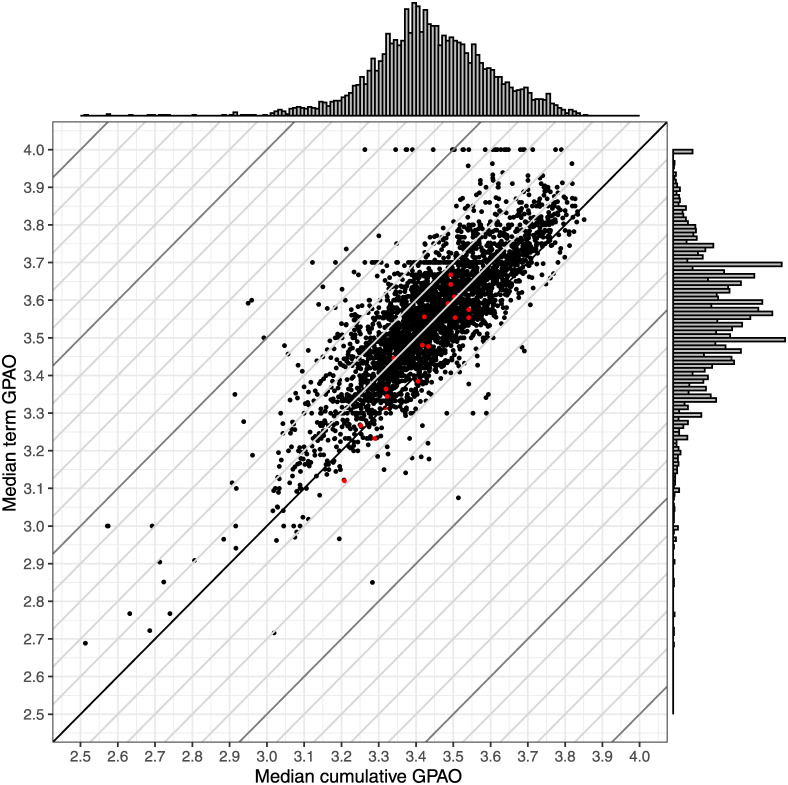
Comparison of term GPAO and cumulative GPAO for over 3,700 courses. Diagonal lines represent the differences between the two GPAO at 0.1 increments. The red points represent the 18 courses explored in detail in the previous sections. The margin plots show the distribution of each GPAO in 0.01 bins. Overall, we find that courses can have different median term and cumulative GPAOs and that term GPAO tends to be the larger of the two.

Fig 5 shows the median cumulative GPAO plotted against the median term GPAO for 3,737 courses at our university. There is a wide variation in both the cumulative and term GPAOs across the university, and at the same time, many of the courses are clustered in the region between GPAOs of 3.3 to 3.8. The distribution the cumulative GPAOs peaks between 3.3 and 3.4 while the term GPAO distribution peaks around 3.5 and 3.7.

When comparing the two measures, the median term GPAO of students in the course is larger than the median cumulative GPAO is in around 90% of the courses. However, the difference between the two GPAOs is typically less than 0.2, or less than the smallest difference between grades on the typical 4.0 scale (0.3). We note that there are cases where the difference between the two measures is greater than 0.5; however, these cases represent a very small percentage of all courses included here (0.3%).

**Fig 6:**
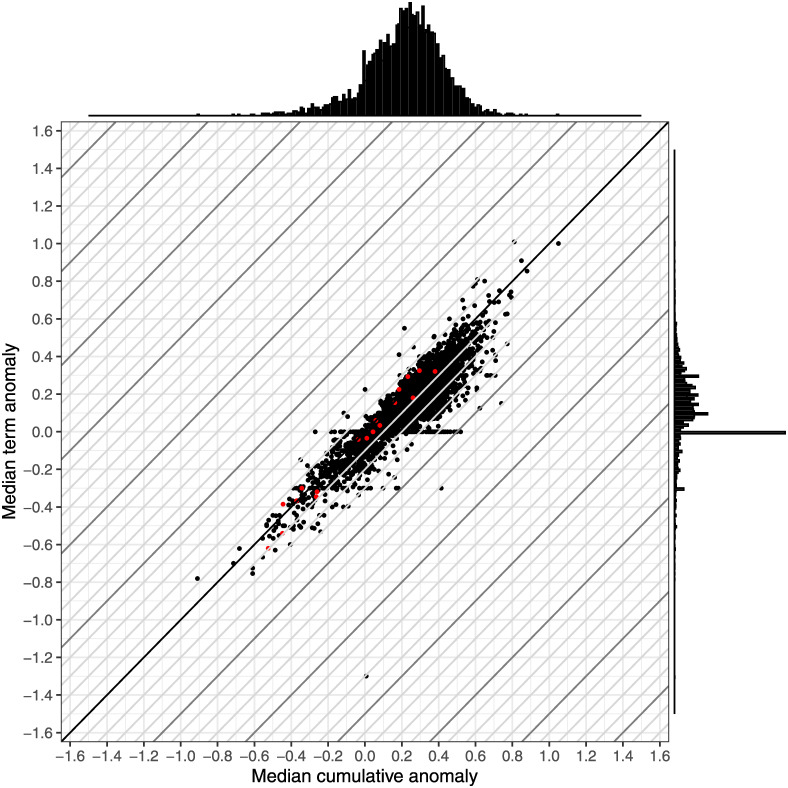
Comparison of term grade anomaly and cumulative grade anomaly for over 3,700 courses. Diagonal lines represent the differences between the two grade anomalies at 0.1 increments. The red points represent the 18 courses explored in detail in the previous sections. The margin plots show the distribution of each anomaly in 0.01 bins. Overall, we find that courses can have different median term and cumulative anomalies and that term grade anomaly tends to be closer to the actual grade.

Focusing on grade anomalies (Fig 6), there is a wide variation in the measures across the courses, though the variation tends to be smaller than that of the GPAOs and the central cluster of courses appears to have a smaller width. Notably, the term anomaly distribution is much more concentrated than that of the term GPAO distribution, now with a single central peak around 0, meaning that the most common result is that the median term GPAO approximately matches the median final grade in the course. Despite being the most common result, only about one in seven courses actually had this result.

In terms of comparing the two measures, the median anomaly is positive in most cases, meaning that the GPAO underpredicts the course grade for a typical student. We do notice some variation in terms of which anomaly is more accurate. If we are concerned with size alone, we find that the median term anomaly is smaller than the median cumulative grade anomaly in nearly 88% of the courses. If we instead consider the distance from zero (i.e., the absolute value), then the median term grade anomaly is smaller than the median cumulative grade anomaly in 81% of the courses.

When comparing the fraction of students outside of a specified threshold for all courses, using the term GPAO results in more students with grade anomalies beyond the threshold. Using the lower bound of the threshold (*θ* = 0.7), 68% of all courses included in the study had more students with grade anomalies beyond the threshold when the term GPAO was used in the calculation compared to the cumulative GPAO. When we used a threshold of *θ* = 1.0 instead, the percent of courses where the term GPAO resulted in more students beyond the threshold increased to 76%.

To account for practical significance, we then required the difference between the fraction of students with grade anomalies beyond the threshold to be different by more than 0.049. In that case, under the *θ* = 0.7 threshold, 59% of the courses did not show a difference while 31% of the courses included in the study had more students with grade anomalies beyond the threshold when the term GPAO was used in the calculation compared to when the cumulative GPAO was used. With a threshold of *θ* = 1.0 instead, the percentages were 81% and 17% respectively.

Finally, in understanding how the choice of GPAO affects conclusions about the grade penalty or grade boost each demographic group experiences, we find the impact to be small. When we required at least one median grade anomaly to be at least 0.099 in magnitude, we found that 7.5% of courses would have a different conclusion about whether the course resulted in a grade boost or grade penalty for a typical student. Of those courses that would have had a different conclusion based on the choice of GPAO, 60% had no more than 200 students enrolled over the 10-year period under study and 84% had no more than 500 students enrolled over the 10-year period.

If we required one median grade anomaly to be at least 0.049 in magnitude instead of 0.099, 12.9% of the courses would have a different conclusion about whether the course resulted a grade boost or grade penalty for a typical student. For the 12.9% of courses that would have had a different conclusion based on the choice of GPAO with 0.049 requirement, those numbers were 56% and 82% respectively. With fewer students enrolled, there would be more noise and variability in the data than for a larger course and hence, we would expect that most course conclusions affected by our choice of GPAO would be for smaller courses.

Considering the 10 demographic groups (by sex, race, first-generation college status, low income status, and transfer student status), we also find limited evidence that the conclusion changes based on the GPAO used in the grade anomaly calculation. Requiring a median grade anomaly be at least 0.099 in magnitude, we find that between 6.3% and 9.7% of the courses would have a different conclusion based on the GPAO used when the results are broken down by demographics. Requiring one median grade anomaly be at least 0.049 in magnitude, those numbers increased to between 10.5% and 14.6%.

Looking into the cases where the conclusion would differ based on our choice of GPAO and the requirement that one median grade anomaly be at least 0.099 in magnitude, we find that it usually happens when the median term anomaly is zero (between 66% and 76% of the courses for the 10 demographic groups) or the median term anomaly is negative (between 20% and 30% of the courses for the 10 demographic groups) and nearly always when the median cumulative anomaly is positive (between 97% and 99% of the courses for the 10 demographic groups).

### Summary of results and answers to our research questions

Here, we address the four research questions our work set out to answer:

*1. How does the term GPAO compare to the cumulative GPAO and, by extension, term grade anomaly to cumulative grade anomaly?* We find that in most cases, these two measures are relatively similar and within 0.2 grade points of each other. However, there are cases where there can be substantial differences between the two.

Across the more than 3,700 courses analyzed in this study, we find that in most of them, the median term GPAO was greater than the median cumulative GPAO. This result is likely a result of low grades only impacting the term GPAO if they occur in the same term as the course of interest while low grades will impact the cumulative GPAO regardless of when they happen in the student’s academic career.

We also found the median term GPAO was a more accurate predictor than the cumulative GPAO was when compared to the student’s final grade for a typical student. However, using the term GPAO instead of the cumulative GPAO resulted in more students with grade anomalies beyond a given threshold, suggesting that while the term GPAO might be better for analyses relying on measures of central tendency like the median, it may not be better for analyses that focus on individual students.

*2. How does the answer to the above question change based on whether the course is an introductory, intermediate, or advanced level course? How does the answer change based on whether the course is a lab or lecture course?* We found all nine lecture courses of interest have larger median term GPAOs than cumulative GPAOs. For the lab courses, the results were mixed, with about half of them having a larger median term GPAO while the others had a larger cumulative GPAO. Due to different trends in lab and lecture courses, we urge caution in aggregating across these types of courses because the effects of GPAO may be different.

Across all courses, we found having a 4.0 GPAO was more common when using the term method than the cumulative method. That is likely because getting a term GPAO of 4.0 means that the student only needs to earn all “A”s in a single semester while getting a cumulative GPA of 4.0 requires the student to earn an “A” in every “other” course they have taken at the university.

Looking at the advanced courses, we found that tails of the GPAO distributions were smaller than those of introductory courses. This finding might reflect the typically higher grades earned by students in advanced courses. The positive median grade anomalies in those courses supports such an interpretation.

Looking at the grade anomalies, we found the lab courses had non-negative median grade anomalies, while the introductory and intermediate lecture courses had negative grade anomalies. These results were true regardless of which GPAO we used. These results imply that, in general, students do better compared to their “other” courses in lab courses than they do in their lecture courses. Given the known difficulty of introductory, large-enrollment STEM courses, it is not surprising that all the introductory courses have negative median grade anomalies, regardless of which GPAO we used. In fact, these courses, along with the intermediate courses in the study, are among the courses at the studied university with the most negative grade anomalies.

In terms of the differences in the anomalies, we find they are relatively small, less than 0.1 grade points in magnitude. This result suggests that, in practice, any results should not be affected by using one GPAO instead of the other. Importantly, we do not find evidence that using one GPAO instead of the other results in a significantly different direction of the median grade anomaly in any of these courses and would affect any conclusions.

Finally, we found there were differences in the tails of the grade anomaly distributions for some courses In those cases, using the term GPAO resulted in longer tails than the cumulative GPAO did. That result was true regardless of which threshold we picked. However, in many of the courses, there were still a relatively large number (> 10% and up to 25%) of students with GPAOs more than 1 grade point different from their final course grade, regardless of which GPAO we used. That is, if researchers want to predict a student’s grade, their grades in “other” courses alone, regardless of whether “other” courses means all other courses taken at the university or in the same term as the course of interest, are not sufficient to do so with high precision. Given that students can earn significantly higher or lower grades for a variety of reasons, we do not consider this a fault of GPAO but rather an inherent limitation of grade prediction using GPAO alone.

Based on our analysis of a subset of courses varied by discipline, level, and format, we do not find consistent evidence that one GPAO should be preferred over the other or that using one or the other would substantially change the results.

*3. RQ3: How do the answers to the above questions change when breaking results down by demographics?* When we break the subset of courses down by demographics, we find mixed results. For nearly every demographic, we do find a difference between the two GPAOs, though the median differences are relatively small—at most, 0.1 grade points. However, whether that difference is statistically significant very much depends on the specific course. Within an individual course, it does appear that the direction of the difference is relatively consistent even though the magnitude varies. Across courses, however, there is not a consistent direction to the results. That is true regardless of whether we are calculating the difference between GPAOs or grade anomalies at the student or course level.

When using effect size instead of statistical significance to evaluate differences, we also did not find any consistent trends. While we did find a few course and demographic pairs with a non-trivial effect size, most of the combinations resulted in an effect size less than 0.1 and hence would be considered trivial.

Across both approaches to examining GPAO by demographics, it does not appear that there should be a clear preference for which GPAO we should use. Once we do pick one of the GPAOs to use, the consistent direction of the differences within a course suggests that we are unlikely to be changing the sizes of effects between groups, but rather, shifting all groups approximately equally.

*4. How does a researcher’s choice of using the term GPAO or the cumulative GPAO in the grade anomaly calculation affect conclusions about the course?* We find limited evidence that we would substantially alter any conclusions based on our choice of GPAO. Based on our requirement of the size of the difference between the grade anomalies, at most around 13% of the courses would have a different conclusion about whether students do better or worse in the course compared to their “other” courses. Even when we split our results by demographics and looked for evidence of different conclusions, we found that at most 15% of courses would be affected. Given that only about 1 in 7 courses would have had a conclusion changed by the choice of GPAO and in most cases, the course had less than 500 students enrolled over a 10-year period, cases where the choice of the GPAO influences the conclusion seem to be the exception rather than the rule.

## Conclusion

Overall, our results suggest that neither term GPAO nor cumulative GPAO is inherently better or should be preferred over the other. Looking across more than 3,700 courses, we find limited evidence that using one or the other would substantially affect a conclusion and establish the upper bound that around one in seven courses could be affected.

For specific research questions, we find that there may be a preference for which GPAO should be used. For student-level grade predictions, the cumulative GPAO may be preferred given the term GPAO does seem more variable, with more students having GPAOs farther from the grade they earned. For analyses with results reported in aggregate at the course level, the term GPAO may be preferred given that we found that using the term GPAO does seem to shrink the anomaly for a typical student in the course. Therefore, we do not recommend one method of calculating GPAO over the other in all cases but instead recommend that researchers consider the data they have and which method of calculating GPAO best aligns with their research questions.

Because we did find small differences in the values of the GPAOs and grade anomalies based on what courses were included in the calculation, we encourage researchers to consider the choice of calculation for GPAO as having a systematic uncertainty. If possible, researchers should perform their analyses with both to determine how sensitive their results are to the choice of GPAO and report how their results are impacted by their choice of GPAO.

## Supporting information

S1 File Alignment with neutral comparison studies. (PDF)

S2 File Results if we use the mean instead of the median. (PDF)

S3 File Grade anomalies over time. (PDF)

## References

[1] Romero C, Ventura S. Educational data mining and learning analytics: An updated survey. WIREs Data Mining and Knowledge Discovery. 2020;10(3):e1355. doi: 10.1002/widm.1355

[2] Du X, Yang J, Shelton BE, Hung JL, Zhang M. A systematic meta-Review and analysis of learning analytics research. Behaviour & Information Technology. 2021;40(1):49–62. doi: 10.1080/0144929X.2019.1669712

[3] Sabot R, Wakeman-Linn J. Grade Inflation and Course Choice. Journal of Economic Perspectives. 1991;5(1):159–170. doi: 10.1257/jep.5.1.159

[4] Ost B. The role of peers and grades in determining major persistence in the sciences. Economics of Education Review. 2010;29(6):923–934. doi: 10.1016/j.econedurev.2010.06.011

[5] Owen AL. Grades, Gender, and Encouragement: A Regression Discontinuity Analysis. The Journal of Economic Education. 2010;41(3):217–234. doi: 10.1080/00220485.2010.486718

[6] Astorne-Figari C, Speer JD. Drop out, switch majors, or persist? The contrasting gender gaps. Economics Letters. 2018;164:82–85. doi: 10.1016/j.econlet.2018.01.010

[7] Astorne-Figari C, Speer JD. Are changes of major major changes? The roles of grades, gender, and preferences in college major switching. Economics of Education Review. 2019;70:75–93. doi: 10.1016/j.econedurev.2019.03.005

[8] Seymour E, Hunter AB. Talking about leaving revisited. Talking About Leaving Revisited: Persistence, Relocation, and Loss in Undergraduate STEM Education. 2019;.

[9] Kugler AD, Tinsley CH, Ukhaneva O. Choice of majors: are women really different from men? Economics of Education Review. 2021;81:102079. doi: 10.1016/j.econedurev.2021.102079

[10] Dekker G, Pechenizkiy M, Vleeshouwers J. Predicting Students Drop Out: A Case Study.; 2009. p. 41–50.

[11] Jayaprakash SM, Moody EW, Lauría EJM, Regan JR, Baron JD. Early Alert of Academically At-Risk Students: An Open Source Analytics Initiative. Journal of Learning Analytics. 2014;1(1):6–47. doi: 10.18608/jla.2014.11.3

[12] Zabriskie C, Yang J, DeVore S, Stewart J. Using machine learning to predict physics course outcomes. Phys Rev Phys Educ Res. 2019;15(2):020120. doi: 10.1103/PhysRevPhysEducRes.15.020120

[13] Yang J, DeVore S, Hewagallage D, Miller P, Ryan QX, Stewart J. Using machine learning to identify the most at-risk students in physics classes. Phys Rev Phys Educ Res. 2020;16(2):020130. doi: 10.1103/PhysRevPhysEducRes.16.020130

[14] Yu R, Li Q, Fischer C, Doroudi S, Xu D. Towards Accurate and Fair Prediction of College Success: Evaluating Different Sources of Student Data; 2020.

[15] Yu R, Lee H, Kizilcec RF. Should College Dropout Prediction Models Include Protected Attributes? In: Proceedings of the Eighth ACM Conference on Learning @ Scale. Virtual Event Germany: ACM; 2021. p. 91–100. Available from: https://dl.acm.org/doi/10.1145/3430895.3460139.

[16] Huberth M, Chen P, Tritz J, McKay TA. Computer-Tailored Student Support in Introductory Physics. PLOS ONE. 2015;10(9):e0137001. doi: 10.1371/journal.pone.0137001 26352403 PMC4564174

[17] Saeidi A, Williams A, Buswell N, Mumm D, Denaro K. Can adding discussion-only active learning increase student learning in materials science class? In: 2018 IEEE Frontiers in Education Conference (FIE); 2018. p. 1–4.

[18] Matz R, Schulz K, Hanley E, Derry H, Hayward B, Koester B, et al. Analyzing the Efficacy of ECoach in Supporting Gateway Course Success Through Tailored Support. In: LAK21: 11th International Learning Analytics and Knowledge Conference. LAK21. New York, NY, USA: Association for Computing Machinery; 2021. p. 216–225. Available from: 10.1145/3448139.3448160.

[19] Koester BP, Grom G, McKay TA. Patterns of Gendered Performance Difference in Introductory STEM Courses. arXiv:160807565 [physics]. 2016;.

[20] Matz R, Koester B, Fiorini S, Grom G, Shepard L, Stangor CG, et al. Patterns of Gendered Performance Differences in Large Introductory Courses at Five Research Universities:. AERA Open. 2017.

[21] Asher A, Silvester K. Evaluating the Effectiveness of Integrated Information Literacy Instruction on Student Outcomes in the English W131 Multilingual Curriculum; 2019.

[22] Weaverdyck N, Anbajagane D, Evrard AE. Differential Assessment, Differential Benefit: Four-year Problem Roulette Analysis of STEM Practice Study. In: Proceedings of the Seventh ACM Conference on Learning @ Scale. Virtual Event USA: ACM; 2020. p. 293–296. Available from: https://dl.acm.org/doi/10.1145/3386527.3406731.

[23] Mead C, Supriya K, Zheng Y, Anbar AD, Collins JP, LePore P, et al. Online biology degree program broadens access for women, first-generation to college, and low-income students, but grade disparities remain. PLOS ONE. 2020;15(12):e0243916. doi: 10.1371/journal.pone.0243916 33306720 PMC7732118

[24] Michael R, Said H. Gendered Performance Differences in Information Technology Courses. In: Proceedings of the 21st Annual Conference on Information Technology Education. Virtual Event USA: ACM; 2020. p. 87–92. Available from: https://dl.acm.org/doi/10.1145/3368308.3415395.

[25] Hayward C, Schulz K, Fishman B. Who wins, who learns? Exploring gameful pedagogy as a technique to support student differences. In: LAK21: 11th International Learning Analytics and Knowledge Conference. LAK21. New York, NY, USA: Association for Computing Machinery; 2021. p. 559–564. Available from: 10.1145/3448139.3448198.

[26] Supriya K, Mead C, Anbar AD, Caulkins JL, Collins JP, Cooper KM, et al. Undergraduate Biology Students Received Higher Grades During COVID-19 but Perceived Negative Effects on Learning. Frontiers in Education. 2021;6:428. doi: 10.3389/feduc.2021.759624

[27] Tarchinski NA, Rypkema H, Finzell T, Popov YO, McKay TA. Extended Exam Time Has a Minimal Impact on Disparities in Student Outcomes in Introductory Physics. Frontiers in Education. 2022;7. doi: 10.3389/feduc.2022.831801

[28] Malespina A, Singh C. Gender differences in grades versus grade penalties: Are grade anomalies more detrimental for female physics majors? Phys Rev Phys Educ Res. 2022;18(2):020127. doi: 10.1103/PhysRevPhysEducRes.18.020127

[29] Irani S, Denaro K. Incorporating Active Learning Strategies and Instructor Presence into an Online Discrete Mathematics Class. In: Proceedings of the 51st ACM Technical Symposium on Computer Science Education. SIGCSE’20. New York, NY, USA: Association for Computing Machinery; 2020. p. 1186–1192. Available from: 10.1145/3328778.3366904.

[30] Jantzer J, Kirkman T, Furniss KL. Understanding Differences in Underrepresented Minorities and First-Generation Student Perceptions in the Introductory Biology Classroom. J Microbiol Biol Educ. 2021;22(3):e00176–21. doi: 10.1128/jmbe.00176-21 34804325 PMC8561840

[31] Dusen BV, Nissen J. HOW STATISTICAL MODEL DEVELOPMENT CAN OBSCURE INEQUITIES IN STEM STUDENT OUTCOMES. JWM. 2022;28(3).

[32] Pearson MI, Castle SD, Matz RL, Koester BP, Byrd WC. Integrating Critical Approaches into Quantitative STEM Equity Work. LSE. 2022;21(1):es1. doi: 10.1187/cbe.21-06-0158 35100005 PMC9250366

[33] Achen AC, Courant PN. What Are Grades Made Of? Journal of Economic Perspectives. 2009;23(3):77–92. doi: 10.1257/jep.23.3.77 20052301 PMC2801426

[34] Evrard A, Schulz K, Hayward C. How Did You Get that A? Selectivity’s Role in Rising Undergraduate Grades at a Large Public University. In: LAK21: 11th International Learning Analytics and Knowledge Conference. LAK21. New York, NY, USA: Association for Computing Machinery; 2021. p. 565–571. Available from: 10.1145/3448139.3448199.

[35] Yeritsyan A, Mjelde JW, Litzenberg KK. Grade Inflation or Grade Increase. Journal of Agricultural and Applied Economics. 2022;54(2):375–393. doi: 10.1017/aae.2022.15

[36] Grove WA, Wasserman T. The Life-Cycle Pattern of Collegiate GPA: Longitudinal Cohort Analysis and Grade Inflation. The Journal of Economic Education. 2004;35(2):162–174. doi: 10.3200/JECE.35.2.162-174

[37] Whitcomb KM, Cwik S, Singh C. Not All Disadvantages Are Equal: Racial/Ethnic Minority Students Have Largest Disadvantage Among Demographic Groups in Both STEM and Non-STEM GPA. AERA Open. 2021;7:23328584211059823. doi: 10.1177/23328584211059823

[38] Boulesteix AL, Lauer S, Eugster MJA. A Plea for Neutral Comparison Studies in Computational Sciences. PLOS ONE. 2013;8(4):e61562. doi: 10.1371/journal.pone.0061562 23637855 PMC3634809

[39] Odden TOB, Marin A, Rudolph JL. How has Science Education changed over the last 100 years? An analysis using natural language processing. Science Education. 2021;105(4):653–680. doi: 10.1002/sce.21623

[40] Odden TOB, Marin A, Caballero MD. Thematic analysis of 18 years of physics education research conference proceedings using natural language processing. Phys Rev Phys Educ Res. 2020;16(1):010142. doi: 10.1103/PhysRevPhysEducRes.16.010142

[41] Westbrook L, Saperstein A. New Categories Are Not Enough: Rethinking the Measurement of Sex and Gender in Social Surveys. Gender & Society. 2015;29(4):534–560. doi: 10.1177/0891243215584758

[42] Bensimon EM. The misbegotten URM as a data point. Los Angeles, CA: Center for Urban Education, Rossier School of Education, University of Southern California. 2016;.

[43] Walden SE, Trytten DA, Shehab RL, Foor CE. Critiquing the “Underrepresented Minorities” Label. In: 2018 CoNECD-The Collaborative Network for Engineering and Computing Diversity Conference; 2018.

[44] Williams TL.’Underrepresented Minority’ Considered Harmful, Racist Language; 2020. Available from: https://cacm.acm.org/blogs/blog-cacm/245710-underrepresented-minority-considered-harmful-racist-language/fulltext.

[45] Teranishi RT. Race, ethnicity, and higher education policy: The use of critical quantitative research. New Directions for Institutional Research. 2007;2007(133):37–49. doi: 10.1002/ir.203

[46] Shafer D, Mahmood MS, Stelzer T. Impact of broad categorization on statistical results: How underrepresented minority designation can mask the struggles of both Asian American and African American students. Phys Rev Phys Educ Res. 2021;17(1):010113. doi: 10.1103/PhysRevPhysEducRes.17.010113

[47] Rask K. Attrition in STEM fields at a liberal arts college: The importance of grades and pre-collegiate preferences. Economics of Education Review. 2010;29(6):892–900. doi: 10.1016/j.econedurev.2010.06.013

[48] Witteveen D, Attewell P. The STEM grading penalty: An alternative to the “leaky pipeline” hypothesis. Science Education. 2020;104(4):714–735. doi: 10.1002/sce.21580

[49] Thompson ME. Grade Expectations: The Role of First-Year Grades in Predicting the Pursuit of STEM Majors for First- and Continuing-Generation Students. The Journal of Higher Education. 2021;92(6):961–985. doi: 10.1080/00221546.2021.1907169

[50] Wasserstein RL, Schirm AL, Lazar NA. Moving to a World Beyond “*p* < 0.05”. The American Statistician. 2019;73(sup1):1–19. doi: 10.1080/00031305.2019.1583913

[51] Rosenthal R, Cooper H, Hedges L, others. Parametric measures of effect size. The handbook of research synthesis. 1994;621(2):231–244.

[52] Cohen J. Statistical Power Analysis for the Behavioral Sciences. 0th ed. Routledge; 1988. Available from: https://www.taylorfrancis.com/books/9781134742707.

[53] Boulesteix AL, Hoffmann S. To adjust or not to adjust: It is not the tests you perform that count, but how you report them; 2022. Available from: https://osf.io/preprints/metaarxiv/j986q/.

[54] Hochberg Y, Tamhane AC, editors. Multiple Comparison Procedures. Wiley Series in Probability and Statistics. Hoboken, NJ, USA: John Wiley & Sons, Inc.; 1987. Available from: http://doi.wiley.com/10.1002/9780470316672.

