# Supplementary material for: How researchers calculate students’ grade point average in other courses has minimal impact
Source: PLoS One. 2023 Aug 18;18(8):e0290109. doi: 10.1371/journal.pone.0290109 (PMC10437965; doi:10.1371/journal.pone.0290109)
Supplement: S1 File — (PDF) [file pone.0290109.s001.pdf]

How researchers calculate students' grade point average in other courses has minimal impact

Alignment with neutral comparison studies

Here, we address how our research choices align with the neutral comparison study criteria set by Boulesteix et al. [1].

A. The main focus of the article is the comparison itself. It implies that the primary goal of the article is not to introduce a new promising method. We are not introducing GPAO or proposing an alternative method for calculating it. Instead, we are comparing two measures that have been used in the literature before.

B. The authors should be reasonably neutral. While some of the authors have published studies using the cumulative GPAO measure, they did not originally propose GPAO and would not benefit if it were found that the cumulative GPAO is the best option to use. As calculating a cumulative GPAO instead of a term GPAO or vice versa does not substantially change the workflow of any research project and amounts to minor analytical changes, none of the authors have an interest in finding that one GPAO is preferable to the other outside of wishing to use evidence-informed best practices when working with measures of prior preparation in their research.

C. The evaluation criteria, methods, and data sets should be chosen in a rational way. Our evaluation criteria are multifaceted, consisting of summary statistics with tests of significance, effect sizes, and distributions. We consider all courses at our university with sufficient data and choose a smaller subset based on common courses and disciplines of interest in previous GPAO papers. As GPAO is a measure of students' grades over time, and researchers would obtain these from a student data warehouse, using data from a student data warehouse as we do is appropriate.

References

[1] Boulesteix AL, Lauer S, Eugster MJA. A Plea for Neutral Comparison Studies in Computational Sciences. PLOS ONE. 2013;8(4):e61562. doi:10.1371/journal.pone.0061562.
