# Supplementary material for: How researchers calculate students’ grade point average in other courses has minimal impact
Source: PLoS One. 2023 Aug 18;18(8):e0290109. doi: 10.1371/journal.pone.0290109 (PMC10437965; doi:10.1371/journal.pone.0290109)
Supplement: S2 File — (PDF) [file pone.0290109.s002.pdf]

How researchers calculate students' grade point average in other courses has minimal impact

Results if we use the mean instead of the median

In our analysis, we have chosen to use the median instead of the mean to summarize the results given that grades are not normally distributed. However, as the mean or average is commonly used to summarize grades, it is not unreasonable to argue that this paper should have used the mean instead of the median. We address that here and show that while using the mean does affect the specifics of our results, the overall conclusions are unchanged.

In the subset of courses, the median term and cumulative GPAOs were always larger than the mean term and mean cumulative GPAO respectively, indicating that the grades distributions are in fact skewed. The difference between the median term GPAO and the mean term GPAO ranged from 0.080 to 0.180 in the 18 courses (0.045 and 0.120 for the difference between the median cumulative GPAO and the mean cumulative GPAO). Looking at the differences between the term and cumulative GPAO in each of the 18 courses, we find that the difference grows when we use the mean instead of the median in 7 of the courses and in the other 11 courses, it decreases. In 8 of the courses, switching from the median to the mean resulted in a change of which GPAO was larger. In all of those cases, the term GPAO was larger using the median rather than the mean while the cumulative GPAO was larger under the mean rather than the median.

While the direction of the gaps between the two GPAO measures changed based on whether we used the mean or the median to summarize the course, the direction of the anomalies were largely consistent. For the term grade anomaly, we found that the direction of the effect (grade penalty or grade boost) only changed for one of the 18 courses when we switched from using the median to using the mean (two courses for the cumulative grade anomaly). For a study looking at grade penalties or grade boosts in the course overall, this result suggests that in practice, using the median or mean to summarize the data should have minimal impact on the conclusions.

When breaking the results down by demographics in the subset of courses, we still found that the direction of the grade anomaly is more or less consistent within each course. To adjust our results for using the mean instead of the median, we used t-tests instead of median tests to determine whether there was a difference between the mean term grade anomaly and the mean cumulative grade anomaly in each of the 18 courses. Which groups and which courses showed a statistically significant difference also did not have a consistent result within subject or demographic group as was the case with using the medians and the median test.

For the effect size analysis, we used Cohen's D and used the standard cutoffs proposed by Cohen of $d \in [0.2, 0.5)$ as a small effect, $d \in [0.5, 0.8)$ as a medium effect, and $d \in [0.8, \infty)$ as a large effect [1]. We acknowledge that these values are arbitrary, but we use them as a useful guide to interpret our results [2]. In doing so, we still found that most demographic groups in most courses would have trivial effect sizes and a few cases of small effect sizes.

Focusing on the larger group of courses, we still found that the term GPAO performed better than the cumulative GPAO. When using the mean term GPAO instead of the median term GPAO, we find that 73% of the courses had larger term GPAOs (recall that 90% of the courses had larger term GPAOs when we used the median). In terms of the grade anomaly, we found that the mean term grade anomaly was smaller than the mean cumulative grade anomaly in 73% of the courses (88% when using the median). When we took the absolute value of the magnitude of the grade anomaly, the mean term grade anomaly was closer to zero than the mean cumulative grade anomaly was in 63% of the courses (81.5% when using the median). We do note however that for the GPAOs and the grade anomalies, their distributions are smoother when using the mean rather than the median (e.g., there is no peak at 0 for the term grade anomaly).

Finally, looking at the fraction of courses where the results could change based on our choice of the GPAO, we find that the percentage is smaller when we used the mean instead of the median in our calculations. That is, using the mean instead of the median would have strengthened our conclusion that the choice of GPAO has limited impact on results. Requiring at least one mean grade anomaly to be at least 0.099 in magnitude, we found that only 2.6% of courses would have had a different conclusion about a grade penalty or grade boost (7.5% when we used the median). Even lowering the minimum size of one of the mean anomalies to be 0.049, only 5.5% of course would have had a different conclusion about a grade penalty or grade boost (12.9% when we used the median).

When breaking the results down by demographics, we find the same results. When we required at least 50 students of the demographic of interest to be enrolled in the course and at least one mean grade anomaly to be at least 0.099 in magnitude, we find that less than 5% of courses would show a different conclusion based on our choice of GPAO (between 6.4 and 9.7% of courses when we used the median). Even with lower requirements on the size of the difference, 0.049, and enrollment in the course, at least 30 students of the demographic of interest, at most 8.2% of courses would have a different conclusion based on our choice of GPAO (14.4% of courses when we used the median with the same lowered conditions).

As the outlier analysis does not use any summary statistics and is unaffected by our choice of using the median or mean, we do not discuss it here.

References

[1] Cohen J. Statistical Power Analysis for the Behavioral Sciences. 0th ed. Routledge; 1988. Available from: `https://www.taylorfrancis.com/books/9781134742707`.

[2] Thompson B. Effect sizes, confidence intervals, and confidence intervals for effect sizes. Psychol Schs. 2007;44(5):423–432. doi:10.1002/pits.20234.
