# Supplementary material for: How researchers calculate students’ grade point average in other courses has minimal impact
Source: PLoS One. 2023 Aug 18;18(8):e0290109. doi: 10.1371/journal.pone.0290109 (PMC10437965; doi:10.1371/journal.pone.0290109)
Supplement: S3 File — (PDF) [file pone.0290109.s003.pdf]

How researchers calculate students' grade point average in other courses has minimal impact

Grade anomalies over time

In the main text, we provide aggregated anomalies over the study duration. Here, we show how the grade anomalies vary in our subset of courses each semester the course was offered. We do this to show how the grade anomalies and the difference between the cumulative grade anomaly and term grade anomaly fluctuate

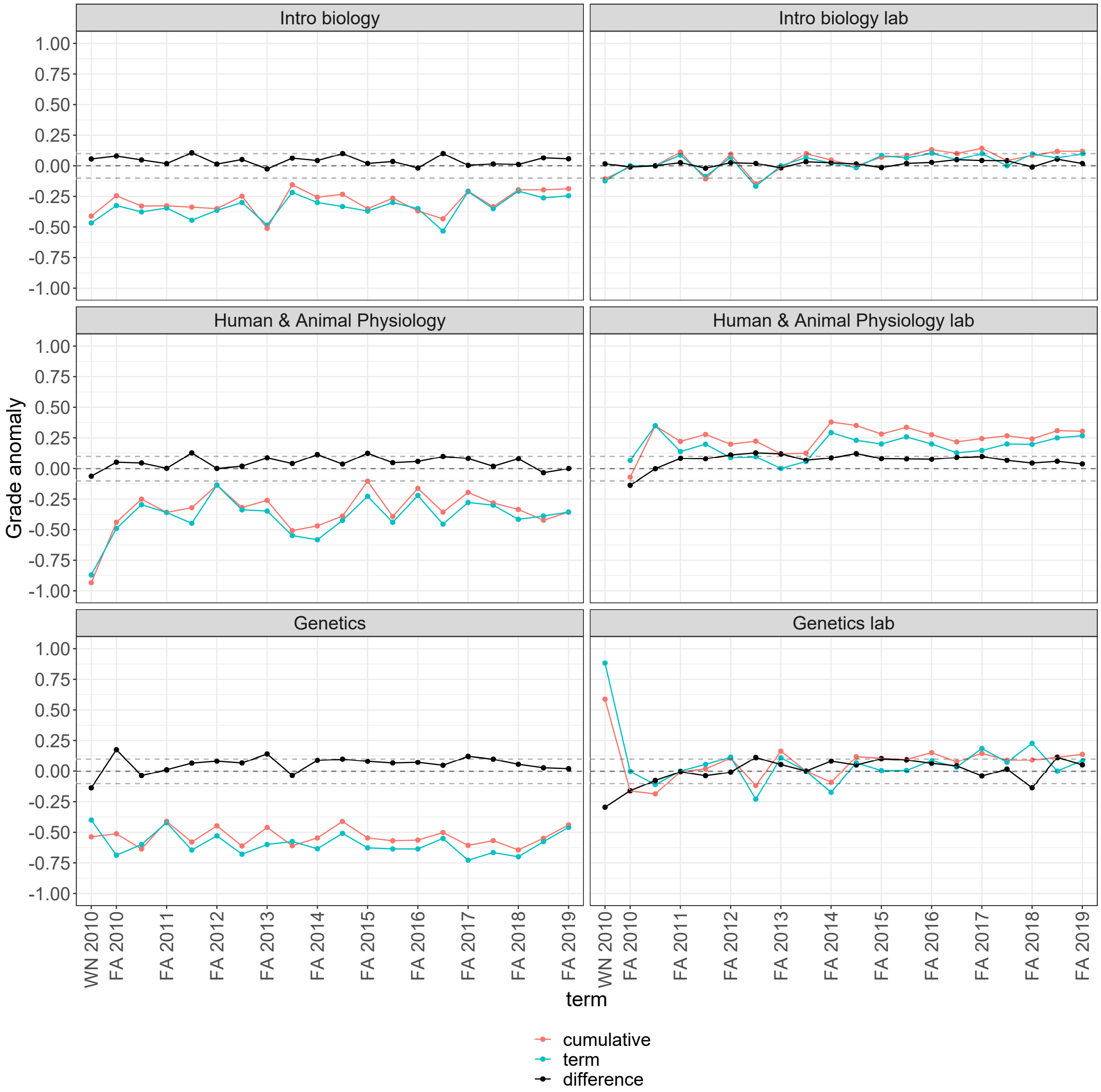

S3 Fig. 1: Comparison of term anomaly and cumulative anomaly in the six biology courses in our subset for each semester the course was offered during the study period along with the difference plotted. The horizontal dashed lines represent a difference of between 0.1 and -0.1, the boundaries of what we have considered a practical difference. While the values fluctuate during the study period, the two anomalies are relatively close.

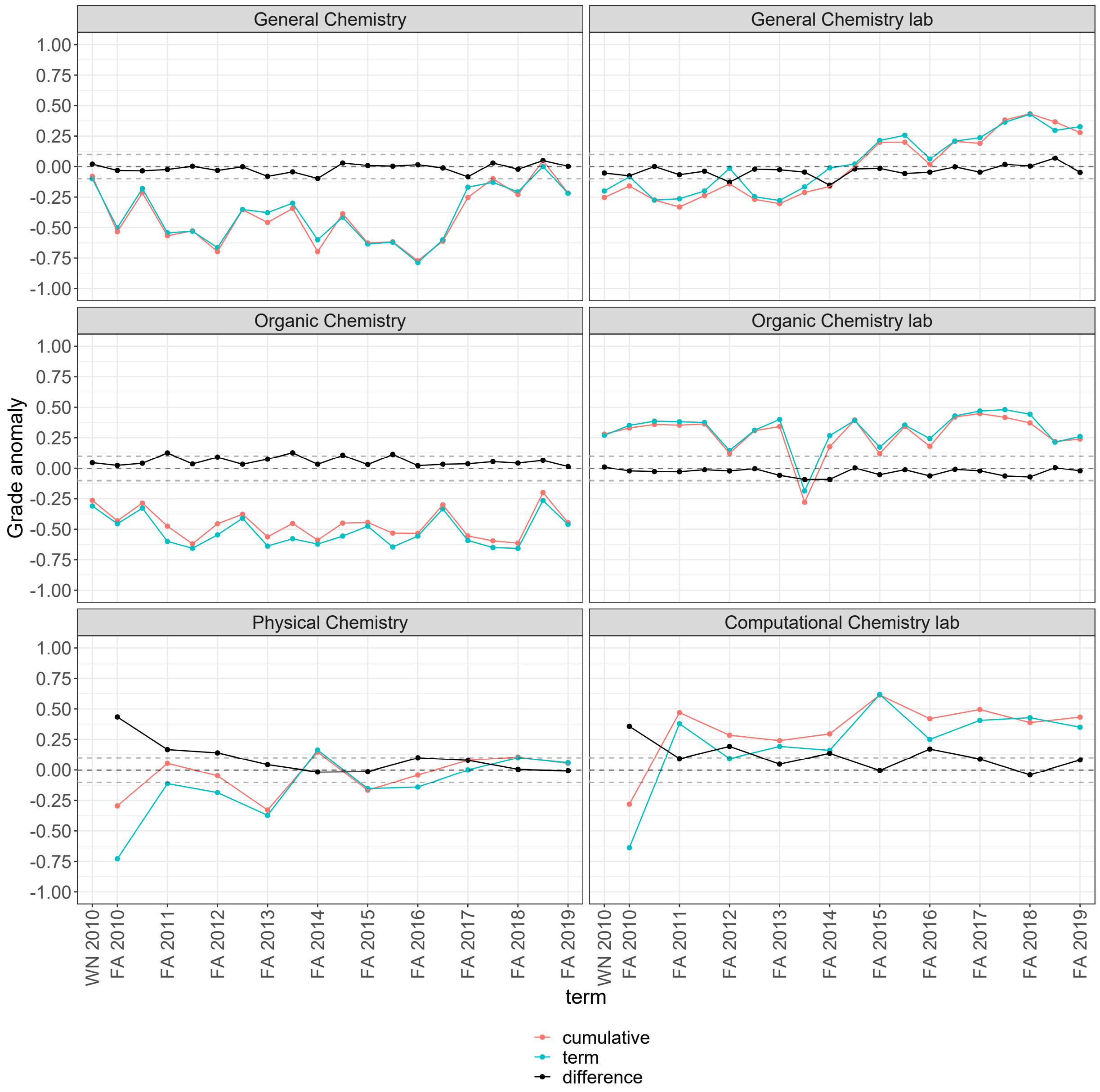

S3 Fig. 2: Comparison of term anomaly and cumulative anomaly in the six chemistry courses in our subset for each semester the course was offered during the study period along with the difference plotted. The horizontal dashed lines represent a difference of between 0.1 and -0.1, the boundaries of what we have considered a practical difference. While the values fluctuate during the study period, the two anomalies are relatively close.

over time, examine whether there may be any long-term variations that challenge our assumption that the data can be aggregated, and compare the variations to our criteria for effect sizes.

Examining first the biology courses (S3 Fig. 1), we notice that the anomalies in the lecture courses vary more than the grade anomalies in the laboratory courses. Yet, in all six biology courses, the difference between the two anomalies tends to be within 0.1 grade points during the two main academic terms. This suggests that even though the term grade anomalies vary, their difference is relatively constant.

Examining the chemistry courses next (S3 Fig. 2), we see a similar pattern, however, the variability in the grade anomalies is closer in scale to that of the lecture courses. At the same time, we still see the general

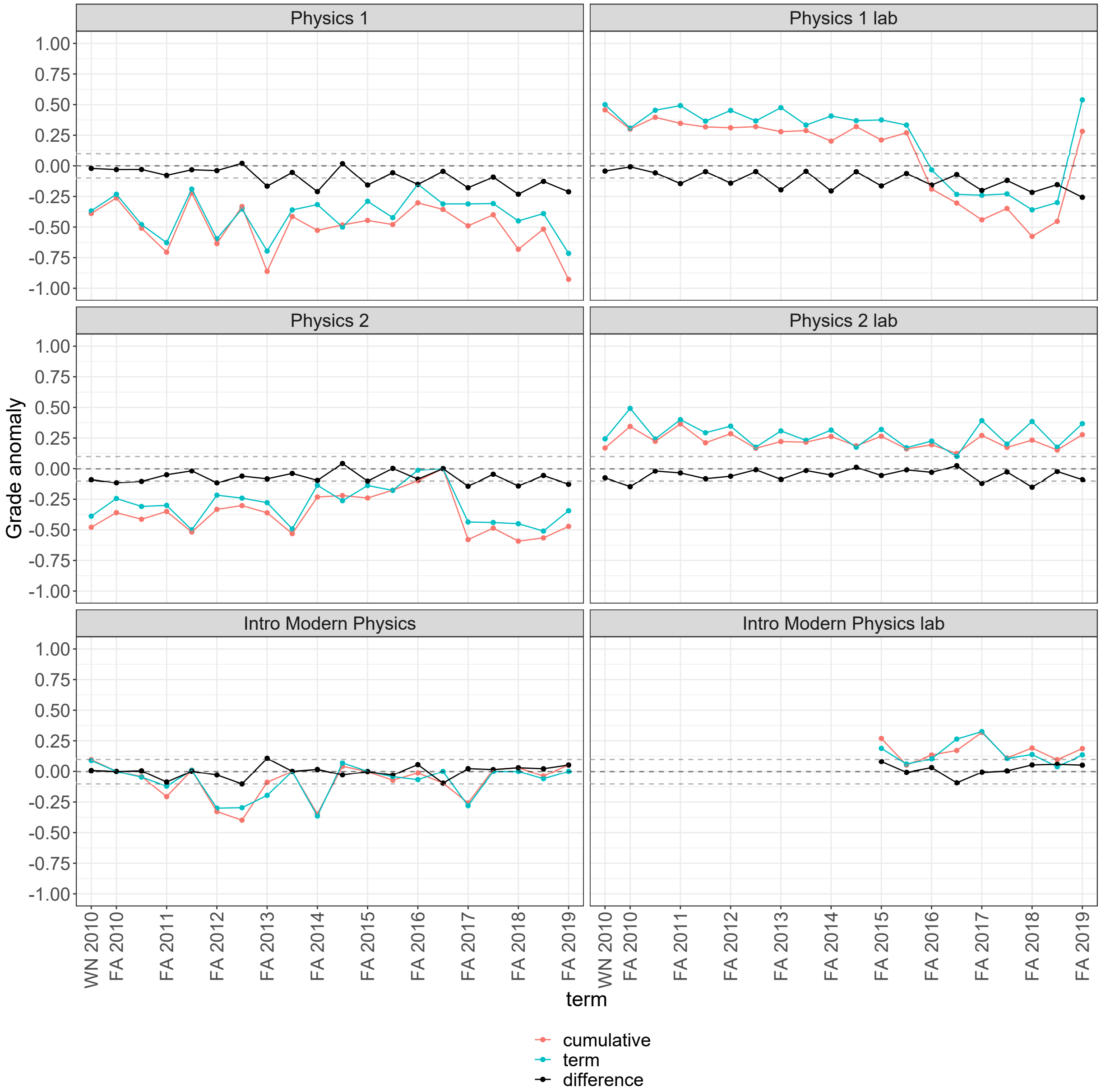

S3 Fig. 3: Comparison of term anomaly and cumulative anomaly in the six physics courses in our subset for each semester the course was offered during the study period along with the difference plotted. The horizontal dashed lines represent a difference of between 0.1 and -0.1, the boundaries of what we have considered a practical difference. While the values fluctuate during the study period, the two anomalies are relatively close.

pattern that most differences in grade anomalies during the fall and winter terms are less than 0.1 grade points.

Finally, examining the physics courses (S3 Fig. 3), we again see a similar pattern, however, the scale of the term variations appears to be smaller. Most of the fall and winter term differences still appear to be within 0.1 grade points of each other with the exception of a few terms.

Overall, the results suggest that while there is term-to-term variation in the grade anomalies, the difference during the traditional academic terms tends to be fairly consistent and variations stay within 0.1 grade points of zero. We find this to be true for around 80% of the academic-year course-term pairs (92%

are within 0.15 grade points). Given these results, we are not concerned about term-by-term variation in the data when aggregating our results.

Based on the direction of the anomaly and our method of GPAO calculation during the fall and winter semesters, the conclusions do not change for any terms in the biology lecture, chemistry lab, physics lecture, or physics lab courses. The conclusions do change in only two terms of the biology lab and chemistry lecture courses under the condition of at least one anomaly being greater than 0.099 in magnitude (as in the main manuscript). That is, across 328 course-term pairs in the fall and winter terms, only 21 (6.4%) would have a different conclusion based on our choice of GPAO about whether the course offers a grade boost or a grade penalty. Of those, 18 course-term pairs have a median term anomaly of zero and one has a median cumulative anomaly of zero. For comparison, in the main manuscript when we aggregated data across ten years, we found that 7.5% of courses would have had a different conclusion about whether the course offered a grade boost or a grade penalty based on our choice of GPAO.
